# Implementing international osteoarthritis treatment guidelines in primary health care: study protocol for the SAMBA stepped wedge cluster randomized controlled trial

**DOI:** 10.1186/s13012-015-0353-7

**Published:** 2015-12-02

**Authors:** Nina Østerås, Leti van Bodegom-Vos, Krysia Dziedzic, Tuva Moseng, Eline Aas, Øyvor Andreassen, Ibrahim Mdala, Bård Natvig, Jan Harald Røtterud, Unni-Berit Schjervheim, Thea Vliet Vlieland, Kåre Birger Hagen

**Affiliations:** National Advisory Unit on Rehabilitation in Rheumatology, Department of Rheumatology, Diakonhjemmet Hospital, PO Box 23, Vinderen, 0319 Oslo, Norway; Department of Medical Decision Making, Leiden University Medical Center, J10-S, P.O. Box 9600, 2300 RC Leiden, The Netherlands; Arthritis Research UK, Primary Care Centre, Research Institute for Primary Care and Health Sciences, Keele University, Keele, ST5 5BG UK; Department of Health Management and Health Economics, Institute of Health and Society, Faculty of Medicine, University of Oslo, Oslo, Norway; Patient Research Panel, Department of Rheumatology, Diakonhjemmet Hospital, PO Box 23, Vinderen, 0319 Oslo, Norway; Department of General Practice, Institute of Health and Society, University of Oslo, Oslo, Norway; Department of Orthopaedic Surgery, Akershus University Hospital, Lørenskog, Norway; Health and Social Services, Nes Municipality, Norway; Department of Orthopaedics, Leiden University Medical Center, J11-S, P.O. Box 9600, 2300 RC Leiden, The Netherlands

**Keywords:** Osteoarthritis, Implementation, Primary care, General practice, Self-management

## Abstract

**Background:**

Previous research indicates that people with osteoarthritis (OA) are not receiving the recommended and optimal treatment. Based on international treatment recommendations for hip and knee OA and previous research, the SAMBA model for integrated OA care in Norwegian primary health care has been developed. The model includes physiotherapist (PT) led patient OA education sessions and an exercise programme lasting 8–12 weeks. This study aims to assess the effectiveness, feasibility, and costs of a tailored strategy to implement the SAMBA model.

**Methods/design:**

A cluster randomized controlled trial with stepped wedge design including an effect, process, and cost evaluation will be conducted in six municipalities (clusters) in Norway. The municipalities will be randomized for time of crossover from current usual care to the implementation of the SAMBA model by a tailored strategy. The tailored strategy includes interactive workshops for general practitioners (GPs) and PTs in primary care covering the SAMBA model for integrated OA care, educational material, educational outreach visits, feedback, and reminder material. Outcomes will be measured at the patient, GP, and PT levels using self-report, semi-structured interviews, and register based data. The primary outcome measure is patient-reported quality of care (OsteoArthritis Quality Indicator questionnaire) at 6-month follow-up. Secondary outcomes include referrals to PT, imaging, and referrals to the orthopaedic surgeon as well as participants’ treatment satisfaction, symptoms, physical activity level, body weight, and self-reported and measured lower limb function. The actual exposure to the tailor made implementation strategy and user experiences will be measured in a process evaluation. In the economic evaluation, the difference in costs of usual OA care and the SAMBA model for integrated OA care will be compared with the difference in health outcomes and reported by the incremental cost-effectiveness ratio (ICER).

**Discussion:**

The results from the present study will add to the current knowledge on tailored strategies, which aims to improve the uptake of evidence-based OA care recommendations and improve the quality of OA care in primary health care. The new knowledge can be used in national and international initiatives designed to improve the quality of OA care.

**Trial registration:**

ClinicalTrials.gov NCT02333656

**Electronic supplementary material:**

The online version of this article (doi:10.1186/s13012-015-0353-7) contains supplementary material, which is available to authorized users.

## Background

Osteoarthritis (OA) is a joint disease characterized by pain, disability, and impaired quality of life. The OA prevalence increases with age and is growing due to the aging of the population and the epidemic of obesity [1]. OA is one of the leading causes of pain and disability for the adult population worldwide [2] and is one of the major contributors to years lived with disability [3]. The costs of treatment and work-related losses are a considerable economic burden [4]. Knee and hip joint replacements for treatment of advanced OA represent a common inpatient surgery. Due to the growing obesity epidemic coupled with aging of the population, the demand for these procedures is expected to accelerate and quadruple by 2030 [5].

Internationally, evidence-based recommendations and standards of care have been developed to improve OA management [2, 6–8]. There is currently no known cure for OA, but non-pharmacological modalities like education, exercise, and weight reduction represent core interventions [2]. The recent national recommendation for imaging in musculoskeletal diseases in general practice recommends conventional radiographs and not magnetic resonance imaging (MRI) in decisions on indication for joint arthroplasty [9]. However, Norwegian and international research indicate that an overall increased access to MRI will result in an increased number of MRI referrals [10], probably also in people with OA.

Structured approaches and stepped care strategies for OA management in order to endorse quality of OA care have been developed in other countries. While Porcheret et al. developed a stepped care strategy for older adults in primary care with knee pain or knee OA [11], the Dutch stepped care strategy (Beating osteoARThritis (BART)) provided a framework for health care providers and people with hip or knee OA to discuss the optimal timing of the various treatment options [12–14]. In the UK MOSAICS study, practice nurses have been trained to provide OA consultations and follow-ups and coordinate referrals to the multidisciplinary care [15]. In Australia, initiatives to reduce the surgery waiting list have been implemented, for example the Osteoarthritis Chronic Care Program (OACCP) in New South Wales. In this programme, a new model of care is being tested, in which a musculoskeletal coordinator assesses individuals and links them with relevant health care providers to support timely and effective care [16]. During 2008, a Swedish project called ‘Better management of patients with OsteoArthritis’ (BOA) was initiated to improve OA care and reduce sick leave among people with OA [17]. The aim of BOA is to offer evidence-based OA information and exercise according to treatment recommendations [2, 6–8] and that surgical interventions should only be considered if non-surgical treatment has been tried and failed. In 2013, a similar project was started in Denmark ‘Good Life with Arthritis in Denmark’ (GLA:D) [18]. Although the health care systems and the models of OA care are somewhat different across countries, the recommendations in the various national and international guidelines are very similar. Thus, aspects of these approaches are likely to be applicable also for other countries.

In the new health sector reform, the Norwegian Directorate of Health has placed the main responsibility for OA treatment in primary health care [19]. However, recent Norwegian research has indicated that people with OA are not receiving the recommended and optimal treatment [20, 21]. This was particularly evident for receipt of OA information, guidance on lifestyle changes, self-management, and weight management. Previous research in general practice has revealed that general practitioners (GPs) are reluctant to discuss weight issues with their patients [22]. A British study on OA monitoring among GPs revealed that the GPs favoured monitoring physical function, pain, and analgesia use over body mass index (BMI), self-management plans, and exercise advice [23]. The authors suggested that the provision of suboptimal care did not result from lack of knowledge and that interventions to improve OA care must address barriers to GPs engaging in optimal care provision [23]. A study among Norwegian physiotherapists (PTs) in private practice showed that exercise treatment was frequently provided but also revealed that the PTs also provided several other treatment modalities with moderate to low quality of evidence or with no evidence from systematic reviews [24].

While good communication is an important element of health care quality, the GP consultation has a limited duration. This implies a challenge for the GPs with respect to patient communication and restricts the amount of information and guidance that can be included within a short consultation. Current OA guidelines recommend physical activity and a healthy lifestyle [2, 6–8]. However, behavioural changes are hard to achieve, and long-term follow-up may be needed. To support for behavioural changes and self-management is a challenge within a GP consultation. A recent systematic review showed that organized follow-up on change of health behaviour can lead to increased level of physical activity [25]. Apart from private physiotherapy, there have up until recently been few other kinds of services that people with OA can be referred to. Frisklivssentral (FLS) (Healthy Living Centre) is a relatively new Norwegian primary health care service that aims to guide and support health-related behavioural changes for individuals with increased risk of, or those who already have, a disease or illness [26]. PTs represent the majority of employees at FLS, but people with other professions may also work at FLS [27]. Close to half of the Norwegian municipalities had established FLSs by 2014. Earmarked subsidies are granted and other incentives for further development are present [28].

Based on international recommendations for OA care and previous research, the SAMBA model for integrated care for people with hip and/or knee OA in Norwegian primary health care has been developed (Additional file 1) and will be implemented in this study. The purpose of the model is to improve quality of OA care in primary health care services by implementing evidence-based international recommendations for OA care among GPs and PTs. An implementation of the model should ensure the provision of optimal, high-quality treatment, and that GPs and PTs deliver the same consistent message to people with OA. However, according to previous research, implementing new care models is challenging, and a systematic review highlighted that there are ‘no magic bullets’ for improving quality of health care [29], but identifying barriers and enablers of implementing guidelines can guide the selection of more effective implementation strategies that are tailored to address those determinants [30].

The barriers and facilitators may be different at different levels of health care [31]. Grol & Wensing have suggested to categorize barriers to and incentives for change among health care professionals into awareness, knowledge, attitude, motivation to change, and behavioural routines [31]. For patients, knowledge, skills, attitude, and compliance are examples of barriers to and incentives for change. Further, barriers and facilitators at the social, organizational, economic, and political context may be present as well [31]. According to Sanders et al., one key obstacle for a successful implementation in primary care may be to achieve ‘coherence’ of the desired practice change with the GPs [32]. GPs may not be able to overcome the inertia of previous practice, may not have the motivation to change, or may not recognize the new approach as distinct from usual practice [33, 34]. Among determinants of guideline use among PTs in primary were care lack of time, poor availability, and limited access to evidence the most important barriers, whereas awareness of and positive attitudes towards guidelines and evidence-based practice, considering guidelines to facilitate practice, and knowing how to integrate patient preferences with guidelines were associated with frequent use of guidelines [35]. In a recent publication, it is stated that one of the major barriers to implement effective management in OA care is suboptimal patient adherence with aspects of the management programme [36].

Interventions tailored to prospectively identified barriers are more likely to improve professional practice than no intervention or dissemination of guidelines or educational materials [37]. Previous research has showed that successful guideline implementation strategies should be multifaceted and actively engage clinicians throughout the process [38]. To identify barriers and facilitators for our OA care model, we performed three focus groups with eight GPs, six PTs, and three patient representatives, respectively (Additional file 2). Based on these discussions, we developed an implementation strategy tailored to the barriers and facilitators identified by the focus group participants (Table 1). The protocol has been reported using the SPIRIT recommendations and the CONSORT guidelines for non-pharmacological interventions [39, 40].

**Table 1:** Multifaceted implementation activities to facilitate the implementation of the SAMBA model for integrated OA care

Target group	Barrier	Activity	Description
Patients	Awareness, knowledge, preference	Education material	All patients receive an OA booklet from the PT with information about OA, treatment, and self-management.
	Awareness, knowledge, compliance	Reminder material, exercise diary	Together with the electronic questionnaire at 3 months, a check-list of recommended OA care will be provided. The patients are asked to keep an exercise diary to register each session.
	Accessibility, availability	Direct access to FLS and PT in private practice, geographically spread PT locations	The PTs working at FLS or in private practice will be asked to prioritize the SAMBA patients by ensuring a quick initial assessment and enrolment in the OA programme. Availability will be ensured by recruiting PTs working at different geographical locations.
General practitioners	Awareness, knowledge, attitude, motivation to change, and behavioural routines	Workshop (provision of information)	The GPs will receive oral and written information on recommended OA care, the PT treatment programme, imaging modalities in OA, and information about the appropriate time to refer to an orthopaedic surgeon. The workshop will be embedded in existing GP meetings, be interactive, and allow time for discussions. SAMBA will be presented as a useful ‘tool’. The PTs will be invited to the GP workshop and vice versa in order to know more about each others’ role in OA care.
	Awareness, knowledge	Education material	The GPs will receive a summary of international guidelines for OA care.
	Awareness, knowledge, attitude, motivation to change, and behavioural routines	Education outreach visits	All general practice clinics will be visited twice during the intervention period. Each clinic will receive a reminder call quarterly by the project coordinator.
	Awareness	Reminder material	Posters, pens, post-it note pads, and mouse mats will be distributed during the workshop.
	Motivation to change	Opinion leaders/endorsement	Local opinion leaders will be identified and asked to promote the intervention among their colleagues. The GPs’ association will be asked to endorse the SAMBA model.
	Awareness, motivation to change	Feedback, audit	Study newsletters will be distributed 3 times a year. Feedback on recruitment rate will be included.
	Accessibility, attitude, behavioural change	Direct access to FLS and PT in private practice	The PT working at FLS or in private practice will be asked to prioritize the SAMBA patients by ensuring a quick initial assessment and enrolment in the OA programme.
Physiotherapists at FLS and in private practice	Awareness, knowledge, attitude, motivation to change and behavioural routines	Workshop (provision of information)	The PTs will be educated in delivering OA care in accordance with clinical guidelines with a standardized patient education material and exercise programme recommendations for patients with OA symptoms primarily from the hip or knee + how to adapt the standard modes of delivery to the needs of the individual OA patient. The PTs will be invited to the GP workshop and vice versa in order to know more about each others’ role in OA care.
	Awareness, knowledge	Education material	The PTs will receive a summary of international guidelines for non-pharmacological OA care.
	Awareness, knowledge, attitude, motivation to change, and behavioural routines	Education outreach visits	All FLSs and private PT practices will be visited twice during the intervention period. Each clinic will receive a follow-up call quarterly by the project coordinator.
	Awareness	Reminder material	Posters, pens, and post-it note pads will be distributed during the workshop.
	Motivation to change	Feedback, audit	Study newsletters will be distributed 3 times a year.
	Awareness, motivation to change	Endorsement, continuing educational points	The Norwegian Physiotherapist Association will be asked to endorse the ActiveA programme and to provide accreditation of the workshop for continuing educational points.

*Dark cells represent intervention periods, and blank cells represent control periods. The inclusion of patients will start on January 15th 2015 and end on June 15th 2016. The last six month follow-up will be in December 2016 and the last 12-month follow-up will be in June 2017

# The 6 municipalities will switch from control phase to intervention phase in a randomized order

### Objective

The main aim of the present study is to assess the effectiveness, feasibility, and costs of a tailored strategy to implement the SAMBA model for integrated OA care in primary health care in Norway.

### Hypotheses

A priori, we hypothesize that the tailored strategy for implementing the SAMBA model for integrated OA care in a primary care setting will:

- Result in higher quality of care than in current OA care (higher compliance with treatment recommendations [2, 6–8] measured by patient-reported achievement of process quality indicators in OA care [20])
- Result in more GP referrals to FLS/physiotherapy and more discharge reports from FLS/PTs to referring GPs than in current OA care
- Result in less GP referrals to MRI for participants with OA and less GP referrals to orthopaedic surgeons in secondary care that does not lead to scheduled joint surgery
- Result in higher patient satisfaction with the new OA care model than in current OA care
- Be more effective than current OA care in improving lifestyle changes (e.g. increase the proportion of people with OA that meet the American College of Sports Medicine’s recommendations for physical activity and reduce the proportion of people with OA that are overweight)

## Methods/design

### Study design

**Fig. 1:**
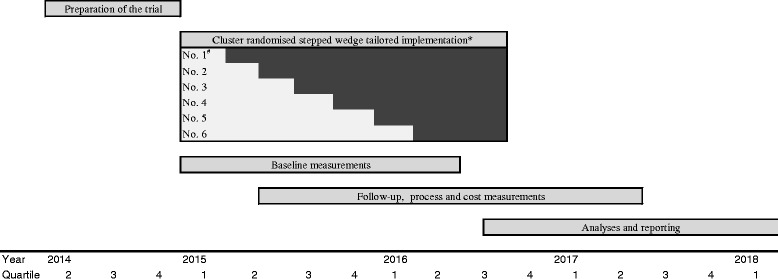
Timeline for the SAMBA project. *Dark cells represent intervention periods, and blank cells represent control periods. The inclusion of patients will start on January 15^th^ 2015 and end on June 15^th^ 2016. The last six month follow-up will be in December 2016 and the last 12-month follow-up will be in June 2017. ^#^The 6 municipalities will switch from control phase to intervention phase in a randomized order

In order to implement the model and to assess the effects of the tailored strategy, a stepped wedge cluster randomized controlled trial will be conducted [41]. The GPs and the PTs in the six participating municipalities (clusters) in Øvre Romerike will switch from control (current OA care) to intervention (implementation of the SAMBA model for integrated OA care) phase in a randomized order. All municipalities start the trial simultaneously and act as controls until the point in time they are randomized to crossover from the control to the intervention phase, and all municipalities have implemented the SAMBA model for OA care by the end of the inclusion period (Fig. 1).

### Setting

The study will be conducted in six neighbouring municipalities north of Oslo (Eidsvoll, Gjerdrum, Nannestad, Hurdal, Ullensaker, and Nes) in collaboration with the regional hospital (Department of Orthopaedic Surgery at Akershus University Hospital), the Department of General Practice at Oslo University Hospital, and National Advisory Unit on Rehabilitation in Rheumatology at Diakonhjemmet Hospital.

### Randomization and blinding

The municipalities will be randomly allocated to one of the six sequences for time of crossover from control to intervention (implementation) phase using a computer-generated list of random numbers. Since the number of inhabitants in the six municipalities varies, stratification on the number of inhabitants less than versus more than 20,000 will be performed to ensure a mix of municipality sizes in the randomized sequence. The allocation sequence will be provided by a statistician. Due to the nature of the implementation strategy, it is not possible to blind the involved GPs and PTs, but the research coordinator performing the telephone interviews will remain blinded. Statistical analyses of primary and secondary outcomes will be performed by the primary investigator and the statistician blinded for group allocation.

### The control phase

During the control phase, the GPs and the PTs receive no activities to promote the use of the SAMBA model for integrated OA care or any other facilitation towards an uptake of the guideline recommendations for OA treatment. Participants with hip and/or knee OA included during the control phase may receive physical therapy as usual, but not the patient education programme, the OA information booklet, exercise diary, or any of the other ‘activities’ in the SAMBA model for integrated OA care prior to 12 months post-baseline.

### The intervention phase (tailored implementation strategy)

Interactive workshops for GPs and PTs will be arranged in the six municipalities successively in close proximity to the time point when they switch from the control to the intervention period. After the workshops, the GPs and PTs will implement the SAMBA model for integrated OA care, which include (1) initial GP consultation; (2) psychotherapist (PT) treatment consisting of 3-h OA patient education programme and 8–12 weeks supervised exercise programme; and (3) GP review consultation followed by self care or a new PT referral or a referral to an orthopaedic surgeon (see Additional file 1).

#### The GP workshop training package

The principal investigator will be responsible for the GP workshops together with the study coordinator, members of the project group, and two orthopaedic surgeons from the regional hospital, Akershus University Hospital. PT representatives will be invited to attend the GP workshop. The GP workshop will last about 1.5 h and consist of three parts: (1) a summary of international evidence-based recommendations for OA care; (2) an introduction to the SAMBA intervention; (3) a presentation by an orthopaedic surgeon on surgical treatment that is provided, the appropriate time to refer people with OA for assessment of surgical treatment, and what kinds of treatment (non-surgical) or other assessments (conventional radiographs) that should have been provided before a referral. The presentation is followed by a discussion with the GPs regarding the timing and the content of referrals for assessment of surgical treatment.

#### The PT workshop training package

The PTs will attend a 1-day workshop-based education programme as part of the ‘Active life with osteoArthritis’ (ActiveA) programme, which is a Norwegian initiative similar to the Swedish BOA [17] (http/:utv.boaregistret.se) and the Danish GLA:D [18] (http/:glaid.dk). The ActiveA programme has been developed and is currently undertaken as collaboration between researchers at Oslo University Hospital and Diakonhjemmet Hospital, Oslo (see Additional file 3 and www.aktivmedartrose.no).

The PT workshop includes education in delivering OA care in accordance with clinical guidelines in a standardized, evidence-based intervention. Education about healthy nutrition and weight reduction will also be included in the workshop. The PTs will receive a complete patient OA education programme (PowerPoint file) to use in their clinical setting as well as a selection of suggested exercises. Furthermore, the PTs will receive information and instructions regarding the SAMBA model for integrated OA care.

### Recruitment of GPs and PTs

GPs and PTs working in private institutes or at FLSs will be invited during outreach visits to each practice and clinic. We aim to recruit 26 (30 %) of the GPs (currently 80 GPs), all PTs (currently 8) at the six FLSs, and 35 (50 %) of the PTs in private practice (currently 70 PTs). In order to facilitate a high attendance among the GPs, the project group will preferably use one of the regular, established meetings in each municipality for the workshop.

### Recruitment of participants with hip and/or knee OA

Participants will be recruited among individuals that visit their GP with activity-related hip or knee pain/complaints. The GPs will identify eligible people with hip and/or knee OA, hand out a single-paged information sheet about the study, and ask for permission for giving the project group their name and phone number. For those who agree, their name and phone number are then mailed by the GP or their secretary in prepaid envelopes to the project group for further contact. The project coordinators will then provide study information by telephone and perform a screening in relation to the inclusion and exclusion criteria (Table 2). Those that comply with the criteria will receive the written information material and consent form and have 2–3 days to decide if they want to participate in the study.

**Table 2:** Criteria for inclusion or exclusion

Inclusion criteria	Exclusion criteria
• Males and females 45 years or older • Activity-related hip and/or knee pain/complaints AND • Clinical signs and symptoms corresponding to hip and/or knee OA OR radiologically diagnosed OA OR registered in the medical journal with the ICPC codes L89 (osteoarthritis of the hip), L90 (osteoarthritis of knee), and/or L91 Osteoarthritis (not classified elsewhere).	• Total hip or knee replacement in the actual joint(s) and no pain/complaints in the other hip or knee joint(s) • Inflammatory rheumatic diseases (e.g. rheumatoid arthritis, spondyloarthritis) • Malignant illness or other major conditions (i.e. unstable cardiovascular disorders or lung disease, dementia) that restrict the ability to adhere to the recommended OA treatment • Do not understand the Norwegian language

A computer programme for identifying eligible people with hip and/or knee OA will be developed. The GP or their secretary may run the programme once a month in order to get a list of patients. This list includes patients that have consulted their GP in the last 30 days and are registered with the following International Classification of Primary Care (ICPC) codes in the past 2 years: L89 (OA of the hip), L90 (OA of the knee), L91 (OA not classified elsewhere), L13 (hip symptoms/complaints), L 15 (knee symptoms/complaints), or L20 (joint symptoms/complaints not classified elsewhere). The programme excludes people registered with ICPC codes L88 (rheumatoid arthritis), T92 (gout), and P70 (dementia). When the GP identify eligible people appearing on this list who were not informed about the SAMBA study during their consultation, a letter will be sent from the GP asking the individuals for permission to be contacted by the study coordinator. Information leaflets and wall posters in the GP waiting rooms as well as newspaper articles and information at local meetings in the Norwegian Rheumatism Association can raise awareness of the study so that the individuals can ask their GP about it. In addition, the PTs at FLS and in private practice will be instructed to ask recently referred people with OA if they have received a request to participate in the SAMBA study.

### Sample size calculations

Previous research in the ‘Musculoskeletal pain in Ullensaker Study’ showed that about 12 % of the adult population self-reported OA in their hip and/or knee joints. Among these, 89 % visited their GPs in the previous year. The estimated adult population (aged 40–70) in the six municipalities in Øvre Romerike is about 35 000, of which about 4200 might have hip and/or knee OA and 3700 might visit their GP during a 1-year period. Based on results from a study in 2012 among 1052 members of the Norwegian Rheumatism Association with hip and/or knee OA, we have estimated the intraclass correlation coefficient (ICC) (using the 19 counties as clusters) to be <0.01. Further, we have estimated that a minimum of 194 individuals in each group among the six clusters with an average of 50 individuals per cluster, achieves 80 % power to detect a 10-unit difference between the group means of the primary outcome measure (OsteoArthritis Quality Indicator questionnaire); when the standard deviation for the primary outcome measure is 24 units, the drop-out rate is 30 %, and the intraclass correlation is 0.01, using a two-sided test with a significance level of 0.05. Hence, we aim to include at least 388 individuals in total.

### The primary outcome measure

The participants with hip and/or knee OA will self-report the primary outcome measure, as well as most secondary outcome measures, at five time points: baseline (shortly after the GP consultation) and then 3-monthly (Table 3). The primary outcome will be patient-reported quality of OA care as measured by the OsteoArthritis Quality Indicator questionnaire, a 17-item questionnaire that includes quality indicators (QIs) related to OA patient education and information, regular provider assessments, referrals, and pharmacological treatment [20]. The items were developed from a literature review of published QIs, expert panels, and patient interviews. The patient self-report questionnaire covers one A4 page with yes/no and ‘not applicable’/‘don’t remember’ as response options, and a previous application has showed acceptable validity and reliability in an OA cohort [20]. The total QI pass rate for each person will be calculated as the total number of QIs they passed, divided by the total number of QIs for which they were eligible. At inclusion, the primary outcome measure may be used to assess the effect of the intervention of the GP consultation, whereas at follow-up, it may be used to assess the total effect of the new model.

**Table 3:** Primary and secondary outcomes

	Measurement scale	Time^a^
Primary outcome measure (patient-reported)		
OsteoArthritis Quality Indicator Questionnaire [20]	0–100 % (pass rate)	0, 3, 6, 9,12
Patient-reported secondary outcome measures		
Pain level in hip/knee past week	NRS 0–10	0, 3, 6, 9,12
Stiffness in the hip/knee past week	NRS 0–10	---“---
Hip/knee function in the past week	NRS 0–10	---“---
Patient global assessment of the OA disease	NRS 0–10	---“---
Patient acceptable symptom state (PASS)	Acceptable/unacceptable	---“---
Function (Knee injury and Osteoarthritis Outcome Score OoL subscale [44]/Hip disability and Osteoarthritis Outcome Score OoL subscale [45] (K/HOOS)	4 items, 5-point scale	---“---
Physical activity (frequency, intensity, duration) [47]	3 items	---“---
Daily hours in sitting position	1 item	---“---
Satisfaction with the care provided (from patient experience questionnaires [48])	1 item, 5-point scale	---“---
Health related quality of life (EQ-5D) [49]	5 items, 5-point scale	---“---
Body weight	kg	---“---
Health care use past 3 months		---“---
Patient Specific Functional Scale [43]	NRS 0–10	Pre- and post exercise programme
Adverse events	1 item	Exercise diary
Measured patient secondary outcomes		
30-s chair-stands test [50]	Number of stands	Pre- and post exercise programme
6-min walk test [51]	Metres	---“---
Stairs test [52]	Seconds	---“---
GP- and PT-reported secondary outcome measures		
Knowledge about recommended OA care	2 items, 5-point scale	Pre- and post-workshop + 6 months post-workshop
Attitude towards OA treatment and recommendations	4 items, 5-point scale	---“---
Behaviour in OA care (referrals, imaging)	---“---	---“---
Register based data secondary outcome measures		
Number of referrals to secondary care that does not lead to scheduled joint surgery		0–12
Number of referrals to MRI for OA assessment		---“---
Number of GP referrals for OA patients to PTs at FLSs/private practice		---“---
PTs at FLSs/in private practice secondary outcome measures		
Number of discharge reports from PTs at FLSs/ private practice to the referring GP		0–12

^a^0 = baseline assessment, the other numbers indicate months after baseline assessment

*MRI* magnetic resonance imaging, *NRS* numeric rating scale

### Secondary outcome measures

Secondary outcomes will include both process and effect evaluation measures reported by the participants with OA, the GPs, and the PTs as well as data obtained from registers (Table 3). The number of ‘responders’ in the two groups will be compared using the OMERACT-OARSI responder criteria [42]. A participant will be classified as a responder if one of the following is fulfilled:

1. High improvement in pain or function
  ≥50 % improvement + absolute change of ≥2 in self-reported pain (numeric rating scale (NRS), 0–10), OR
  ≥50 % improvement + absolute change of ≥2 in self-reported function (NRS, 0–10)
2. Improvement in at least two of the three following:
  ≥20 % improvement + absolute change ≥1 in self-reported pain (NRS, 0–10)
  ≥20 % improvement + absolute change ≥1 in patient global assessment of disease activity (NRS, 0–10)
  ≥20 % improvement + absolute change ≥1 in self-reported function (NRS, 0–10)

Only participants recruited during the intervention phase will keep an exercise diary. Their attendance at patient OA education sessions, exercise sessions (group/ individual), healthy eating programme (FLS) as well as use of the number of exercise sessions recorded in the exercise diary will be registered. The 6-min walk test, stair test, and 30-s chair-stand test will be administered by the PTs before and after the treatment period. Test results are registered in the exercise diary by the PT. In collaboration with the PT, the participants fill in the Patient Specific Functional Scale [43] by self-selecting between one and three activities that they are unable to do or have difficulty doing as a result of their OA disease. The level of difficulty for each activity is patient-reported before and after the treatment period in the exercise diary using an 11-point numeric rating scale (0 = unable to perform activity, 10 = able to perform activity at pre-disease level).

GPs’ and PTs’ attendance at the workshops will be registered. The GPs and PTs will respond to a questionnaire immediately before and after the workshop as well as after 6 months. Telephone interviews with estimated 10 % of the involved GPs and PTs will be undertaken 6 months post-workshop regarding whether they have implemented the SAMBA model (i.e. referred to physiotherapy, arranged patient OA education programmes, offered a supervised exercise programme), their experiences with the implementation strategy, and reasons for eventual non-use of the SAMBA model. Also, about 10 participants with hip and/or knee OA will be telephone interviewed regarding their experiences with the SAMBA model for integrated OA care. The interview data will be explored by text analyses, coded, and combined into broad categories.

### Data collection

The participants with hip and/or knee OA will be asked to complete electronic questionnaires via a link sent by email. If a participant has no email address or internet access or is reluctant to reply electronically, the questionnaire and a prepaid envelope will be mailed to the participant. Information on participant age, gender, joint replacements, and comorbidity will be collected by the project coordinators during the telephone screening. Other participant characteristics and OA disease related information will be self-reported at baseline (body height, marital status, years of education, occupational status, joints with OA, most affected joint, years with OA diagnosis, and function (Knee injury and Osteoarthritis Outcome Score/ Hip disability and Osteoarthritis Outcome Score ADL subscale)). Demographic data on the GPs and PTs will be collected at the workshop (age, gender, years since graduation, speciality, type of practice, number of GPs/PTs in the practice, number of weekly working hours, and number of people on the GPs’ lists).

### National register data

We will apply for permission to extract data on prescription of medication, sick leave, secondary health care utilization, and total joint surgery from national registers (i.e. The Norwegian Prescription Database, the register of The Norwegian Labour and Welfare Administration, The Norwegian Patient Register, The Norwegian Arthroplasty Register) to merge with collected data after the study has ended. Consent to data merging will be obtained from the participants with hip and/or knee OA.

### Cost-utility

Based on the findings in this study, two economic evaluations will be conducted, applying both health system and societal perspectives. Costs in the health care sector comprise intervention costs and costs related to treatment or assessments, while societal costs include production loss, as well as costs for the individuals and the family. The primary economic evaluation will be cost-utility analysis (CUA) of the incremental cost per quality adjusted life years (QALYs) gained, which will be calculated using the EQ-5D scores at baseline, 3, 6, 9 and 12 months. The secondary evaluation will be a cost-effectiveness analysis (CEA) based on a disease-specific measure of function: Knee injury and Osteoarthritis Outcome Score OoL subscale [44]/ Hip disability and Osteoarthritis Outcome Score OoL subscale [45] (K/HOOS). Incremental cost per K/HOOS will be calculated as the ratio of the difference between groups in mean cost to the difference in mean K/HOOS. By means of bootstrapping, cost-effectiveness acceptability curves (CEACs) will be used to consider the uncertainty surrounding the cost-effectiveness of the integrated OA care model by plotting the probability that the model is cost-effective according to a range of willingness-to-pay thresholds.

### Statistical analyses

Baseline characteristics of the participants with OA, the GPs, and the PTs will be presented for the total study and stratified by cluster. Participant characteristics will also be presented stratified by group allocation. The primary effect analysis will be performed on an intention to treat basis by comparing OA QI summary pass rate in the control group vs. the intervention group with the 6-month follow-up as the primary evaluation time point. Multilevel mixed-models will be fitted to adjust for the effect of clustering (municipality), participant (patient), and repeated measures over time. Analyses of effect and process outcomes in regression analyses will include the baseline value of the outcome variable and be adjusted for covariates at the individual patient participant level (age, gender, education level, pain (NRS 0–10), BMI, baseline function (K/HOOS ADL), self-reported comorbidity). To adjust for potential differences related to GPs behaviour, we will include attendance at the GP workshop as a covariate in analyses. A CACE analysis will be performed to provide an unbiased estimate of treatment effect for participants treated as per protocol specification (in the intervention group: participants having attended the OA patient education programme and performed an exercise programme for at least 8 weeks).

### Ethical approval

The Regional Committee for Medical and Health Research Ethics decided that ethical approval was not required under the Norwegian Act on medical and health research for this type of study (ref. no: 2014/1739 REK south-east C). The study is in accordance with the Personal Data Act and the Personal Health Data Filing System Act as approved by the Data Inspectorate/Data Protection Official on December 22, 2014. The data collection will be conducted in compliance with Good Clinical Practices protocol and the Declaration of Helsinki principles. The participants with OA will receive written and oral information about the study, and written informed consents will be obtained prior to baseline data collection.

### Trial status

The preparation of the study components and the recruitment of GPs and PTs were completed in December 2014. The SAMBA study data collection started in January 2015 and is currently (October 2015) ongoing.

## Discussion

This study aims to implement the SAMBA model for integrated OA care aiming to improve professional practice and patient outcomes and reduce non-desired events (e.g. unnecessary referrals to secondary care, unnecessary use of costly imaging modalities, use of treatment modalities supported by low quality of evidence). The model will be implemented and evaluated in primary health care in six municipalities in a stepped wedge cluster randomized controlled trial.

This study represents a collaborative project including six municipalities, one hospital department, and two national research environments aiming to fulfil the intentions of the Norwegian Health Care Coordination Reform. [46] The new OA care model meets the current need for a multidisciplinary approach to manage people with chronic diseases and strengthen the health care services through collaboration while keeping the individual in focus. To ensure feasibility and compliance both patient representatives, GPs and PTs will be actively involved in the model development and implementation. The study also includes close collaboration with international experts, which will contribute in the study and share relevant experiences from similar implementation studies.

This large cluster stepped wedge randomized trial will add to the current knowledge on structured approaches aiming to improve the uptake of evidence-based OA care recommendations in primary health care, which may improve the quality of OA care. The study may provide new knowledge that can be used in national and international implementation initiatives designed to improve the quality of OA care.

## Additional files

Additional file 1:
**The SAMBA model for integrated OA care.**This file contains a brief description of the development of the SAMBA model for integrated OA care and the workshop training packages. The GP and PT intervention is described more in detail, and the SAMBA model is illustrated in a figure. (DOCX 32kb) Additional file 2:
**Focus group interviews.**This file contains the results from three focus group interviews aiming to identify potential barriers and facilitators for the SAMBA model implementation and the workshop training packages. (DOCX 12kb) Additional file 3:
**Brief overview of The SAMBA/ActiveA physiotherapist workshop training package and the patient management programme.** This file contains an overview of the SAMBA/ActiveA physiotherapist workshop training package and the patient management program, which is illustrated in a model. Exercises for improving neuromuscular control, muscle strength and range of motion are illustrated. (DOCX 832kb)

## Abbreviations

ActiveA: active life with osteoArthritis
BMI: body mass index
FLS: Frisklivssentral (Healthy Living Center)
GP: general practitioner
K/HOOS: Knee injury and Osteoarthritis Outcome Score/ Hip disability and Osteoarthritis Outcome Score
MRI: magnetic resonance imaging
NRS: numeric rating scale
OA: osteoarthritis
PASS: patient acceptable symptom state
PT: physiotherapist
QALY: quality adjusted life years
QI: quality indicator
