# Supplementary material for: Implementing international osteoarthritis treatment guidelines in primary health care: study protocol for the SAMBA stepped wedge cluster randomized controlled trial
Source: Implement Sci. 2015 Dec 2;10:165. doi: 10.1186/s13012-015-0353-7 (PMC4668617; doi:10.1186/s13012-015-0353-7)
Supplement: Additional file 1: — The SAMBA model for integrated OA care.This file contains a brief description of the development of the SAMBA model for integrated OA care and the workshop training packages. The GP and PT intervention is described more in detail, and the SAMBA model is illustrated in a figure. (DOCX 32kb) [file 13012_2015_353_MOESM1_ESM.docx]

**Additional file 1. The SAMBA model for integrated OA care.**

**Development of the SAMBA model for integrated OA care and the workshop training packages**

The model and the workshop training packages were developed by a local reference group and the project steering group based on experiences from previous research trials in the Netherland and the UK. Drafts for the model and workshop training packages were presented at three focus groups. The local reference group consists of 1 GP, 2 PTs working at FLSs, 1 PT working in private practice and 1 patient research partner. All six municipalities and all professions were represented in the group, which was established to ensure relevance, feasibility and local compatibility for the intervention. The Project Steering Group consists of a professor/physiotherapist, a professor/medical doctor, a director of an Orthopaedic clinic/orthopaedic surgeon, a senior researcher/physiotherapist, a municipality chief medical officer, a study coordinator and a patient research partner. Brief summaries of international recommendations for pharmacological and non-pharmacological OA care were written by the project leader and the study coordinator with comments from the steering group. The final version of the model and the workshop training packages were developed after receiving comments and suggestions from the focus groups (see Additional file 2).

**The SAMBA model for integrated OA care**

The model includes implementation of a standardised, evidence-based intervention intending to facilitate an active and healthy lifestyle among people with OA. The model aims to increase the collaboration between health care professionals within and across health care levels to ensure that people with OA will experience timely, well integrated, and high quality care.

*The GP intervention*

During the initial consultation, the GP will talk with the participant about the OA diagnosis and provide information, advices and treatment in accordance with evidence based current recommendations for OA care[1-4]. The GP will emphasize that information, exercise and weight reduction represent the recommended core treatment in current guidelines, and make clear that physical activity is safe and beneficial for people with OA. The GP will refer eligible participants to 8-12 weeks PT treatment in private practice or at FLS. The GP will schedule a follow-up after the treatment period and will receive a treatment report from the PT. Depending on the participant’s outcomes, experiences, and motivation, the GP and the participant may decide on a new treatment referral, or that the participant can be supported with self management, or on a referral to secondary care for assessment of surgical treatment.

*The PT intervention (patient management program)*

This intervention will include an initial assessment with individual examination, functional testing and goal setting. The PTs will arrange patient OA education sessions regularly providing patient education on the OA disease, and encouraging self-management, healthy eating, exercising and maintaining an active lifestyle (see Additional file 3). Participants will be invited to take part in exercise groups, with individually adapted exercise programs. For most participants the primary focus of these exercises will be to improve neuromuscular control and lower extremity muscular strength. Participants should attend two group exercise classes each week, for eight to twelve weeks. The participants will be monitored closely with regularly adjustments of the exercise programs to facilitate optimal individual progression. The PTs will follow the American College of Sports Medicines (ACSM) guidelines for healthy adults[5] for prescription of individually tailored exercise programs. In addition PTs will encourage participants to accomplish a third weekly exercise session focusing on aerobic capacity.

Before and after the exercise period the PTs will perform simple field tests of physical fitness on all participants. The included tests are 30-s chair-stand test, six-minute walk test and a stair test [6-8]. The participants will be motivated to maintain regular physical activity. A brief discharge report from the PT will be sent to the referring GP.

At the FLS, the therapeutic intervention is based on principles for motivational interviewing[9]. The participants will receive structured individualized goal setting process and support on lifestyle change. The FLS intervention aims to prepare participants for physical activity and healthy eating by preventing relapse, increasing self efficacy, solve ambivalence, and maintain supportive self talk. Individualized plans for maintained physical activity will be developed. When appropriate, the participants can additionally attend the ‘Healthy eating’ and/or ‘Self-management’, which represents group education programmes run by the PTs at the FLS lasting ten hours each.

*OA information booklet and the exercise diary*

Participants included in the invention phase will receive an information booklet with information about OA, treatment and self-management from their PT. This booklet will reinforce verbal information given by PT during the patient OA education sessions and will also serve as information source should uncertainties arise in the future. The participants in the intervention group will be asked to keep an exercise diary that include a registration of the exercise session date, the duration of strength and/or aerobic exercises in each session and the intensity using the Borg Scale for ratings of perceived exertion. The diary also contains information on exercise in OA and explains that it is safe and beneficial to exercise and be physically active.

**The SAMBA model for integrated OA care**

**FLS**

- Patient OA education (3hrs)
- Exercise programme (8-12 weeks)
- Healthy eating programme

**GP consultation**

- Information on the disease, treatment alternatives and recommended OA care
- Refer to PT

Self-management

Refer to secondary care for assessment of surgical treatment

Refer to PT/FLS for extended supervised exercise programme

**GP review**

**consultation**

- Review treatment report from PT and the patient’s own experiences
- Discuss self-management and eventual new referrals

**Private PT**

- Patient OA education (3hrs)
- Exercise programme

(8-12 weeks)

**References**

1. National Clinical Guideline Centre. Osteoarthritis. Care and management in adults. London (UK): National Institute for Health and Care Excellence (NICE); 2014 Feb. 36 p. (Clinical guideline; no. 177).

2. Hochberg MC, Altman RD, April KT, Benkhalti M, Guyatt G, McGowan J et al. American College of Rheumatology 2012 recommendations for the use of nonpharmacologic and pharmacologic therapies in osteoarthritis of the hand, hip, and knee. Arthritis Care Res (Hoboken). 2012;64:455-74.

3. Fernandes L, Hagen KB, Bijlsma JW, Andreassen O, Christensen P, Conaghan PG et al. EULAR recommendations for the non-pharmacological core management of hip and knee osteoarthritis. Ann Rheum Dis. 2013;72:1125-35. doi:10.1136/annrheumdis-2012-202745.

4. McAlindon TE, Bannuru RR, Sullivan MC, Arden NK, Berenbaum F, Bierma-Zeinstra SM et al. OARSI guidelines for the non-surgical management of knee osteoarthritis. Osteoarthritis Cartilage. 2014;22:363-88. doi:10.1016/j.joca.2014.01.003.

5. Garber CE, Blissmer B, Deschenes MR, Franklin BA, Lamonte MJ, Lee IM et al. American College of Sports Medicine position stand. Quantity and quality of exercise for developing and maintaining cardiorespiratory, musculoskeletal, and neuromotor fitness in apparently healthy adults: guidance for prescribing exercise. Med Sci Sports Exerc. 2011;43:1334-59. doi:10.1249/MSS.0b013e318213fefb.

6. Jones CJ, Rikli RE, Beam WC. A 30-s chair-stand test as a measure of lower body strength in community-residing older adults. ResQExercSport. 1999;70:113-9.

7. American Thoracic S. ATS statement: guidelines for the six-minute walk test. AmJRespirCrit Care Med. 2002;166:111-7.

8. Cataneo DC, Cataneo AJ. Accuracy of the stair climbing test using maximal oxygen uptake as the gold standard. J Bras Pneumol. 2007;33:128-33.

9. Miller WR, Rollnick S. Ten things that motivational interviewing is not. BehavCogn Psychother. 2009;37:129-40.
