# Supplementary material for: Implementing international osteoarthritis treatment guidelines in primary health care: study protocol for the SAMBA stepped wedge cluster randomized controlled trial
Source: Implement Sci. 2015 Dec 2;10:165. doi: 10.1186/s13012-015-0353-7 (PMC4668617; doi:10.1186/s13012-015-0353-7)
Supplement: Additional file 2: — Focus group interviews.This file contains the results from three focus group interviews aiming to identify potential barriers and facilitators for the SAMBA model implementation and the workshop training packages. (DOCX 12kb) [file 13012_2015_353_MOESM2_ESM.docx]

**Additional file 2. Focus group interviews.**

**Focus group interviews to identify possible barriers and facilitators for the SAMBA model implementation and the workshop training packages.**

Three focus groups with 8 GPs, 6 PTs and 3 patient representatives, respectively, were performed to identify barriers and facilitators for the new model and the intervention. The GPs represented two general practices in two of the municipalities in the study. The PT represented private PTs from one institute and three of the FLS in the study municipalities. Two of the patient representatives were recruited from one of the municipalities, and one representative is a member of the Patient Research Panel at Diakonhjemmet Hospital.

Amongst other barriers raised during the GP focus group were lack of motivation (non-attendance at meetings/seminars/workshops), time commitment/restrictions (busy work days), poor accessibility to PT treatment (long waiting-lists) and that people with OA may displace other people from PT treatment. Furthermore, one of the GPs said that they usually do not know what the PTs include in their treatment sessions, nor do they receive a PT treatment report, and that people with OA may perceive no need for or be resistant towards recommended care (i.e. do not want to do exercises, demand magnetic resonance imaging instead of conventional radiographs). The GPs suggested to tailor make and embed the workshop into regular GP meetings, present the SAMBA intervention as a ‘tool’ for the GPs that does not lead to extra workload, provide a presentation of the FLS and about the PT treatment for the GPs and highlight that the program is evidence-based. The GPs thought that increased awareness and knowledge about non-recommended treatment and imaging modalities would decrease the use of these (i.e. provide updated knowledge to the discussion with people with OA about the need to do an MRI).

The PTs at the FLS mentioned that they have limited time for individual treatment as most activities are group based (with possibilities to individually adjust the exercise dosage). The FLS is a relatively new service with limited resources, and the group exercise sessions need be open for people with other diagnoses. People with OA that experience severely reduced functional ability may benefit more from treatment by PTs in private practice due to potentially more opportunities for individual follow-up and adjustments. The PTs in private practice often have long waiting lists, which represent a barrier for the study logistics. In the small municipalities, PTs at the FLS and in private practice can collaborate on patient education in order to recruit enough participants for running education sessions at a regular interval.

The patient focus group raised that long distances and limited public transport might be a barrier for adherence to the treatment program at FLS or in PT private practice for those that do not have or drive a car. They also mentioned the long waiting lists for PTs in private practice as a potential barrier for the study. Furthermore, they suggested that an exercise diary would motivate participants to adhere to the exercise program.
