# Supplementary material for: Implementing international osteoarthritis treatment guidelines in primary health care: study protocol for the SAMBA stepped wedge cluster randomized controlled trial
Source: Implement Sci. 2015 Dec 2;10:165. doi: 10.1186/s13012-015-0353-7 (PMC4668617; doi:10.1186/s13012-015-0353-7)
Supplement: Additional file 3: — Brief overview of The SAMBA/ActiveA physiotherapist workshop training package and the patient management programme. This file contains an overview of the SAMBA/ActiveA physiotherapist workshop training package and the patient management program, which is illustrated in a model. Exercises for improving neuromuscular control, muscle strength and range of motion are illustrated. (DOCX 832kb) [file 13012_2015_353_MOESM3_ESM.docx]

**Additional file 3. Brief overview of The SAMBA/ ActiveA physiotherapist workshop training package and the patient management program**

1. The physiotherapist educational program is a one day (9 hours) seminar which includes theoretical knowledge about osteoarthritis (OA), scientific evidence for using patient education, exercises and weight loss as the first choice of treatment for OA patients, information about the “ActiveA” program (organization, inclusion/exclusion criteria, extent of the program, the intervention etc.), and practical training in the exercise program and reviewing the material of the OA patient-education. At the end of the seminar the participants will receive accreditation as an “ActiveA physiotherapist”
2. The patient management program: The program includes a 3 hours patient-education program and a semi-standardised individually tailored exercise therapy program performed individually or in groups. The structured education program include information about OA (symptoms and signs, risk factors, treatment etc.), the importance of physical activity, appropriate activity and training modalities, self-management and sharing experiences with peers living with OA. The exercise therapy program is based on the BOA and GLA:D projects and previously published exercise programs from RCTs for lower limb[1, 2]. The program includes a warm-up session, functional exercises, strengthening exercise and flexibility exercises. The exercise program focus on the lower limbs and consist of exercises in both open and closed chain and are performed both with and without the use of external loading. In every exercise there is a focus on high neuromuscular control during the performance. The program is individually tailored, but it is encouraged that patients perform their training sessions together with other patients under supervision of an AktivA-trained physiotherapist. They are also encouraged to perform home exercises/activities with a supplementary focus on aerobic capacity. Intensity is based on The American College of Sports Medicine Guidelines for Developing and Maintaining Cardiorespiratory, Musculoskeletal, and Neuromotor Fitness in Apparently Healthy Adults[3]. Progression is monitored by the physiotherapist and exercises are individually adjusted throughout the exercise period. The program lasts for a minimum of 8 weeks, after this period the patients are expected to continue their exercise program and physical activities by their own on individual basis or together with peers. Patients who do not want to take part in the group sessions have the option to perform all exercises at home, but they are strongly encouraged to join the groups supervised by a physiotherapist.

After completing the patient education program and the exercise program, the patients who are part of the SAMBA study follows a somewhat different path compared to the ActiveA patients. For more information about the ActiveA program and follow-up, visit [www.aktivmedartrose.no](http://www.aktivmedartrose.no). The SAMBA patients visit their GP after three months for a review. The patient and GP decide together whether self-management is now appropriate, whether there is need for renewed referral to physiotherapy or whether it is appropriate with a referral to secondary health care.

**References**

1. Fernandes L, Storheim K, Nordsletten L, Risberg MA. Development of a therapeutic exercise program for patients with osteoarthritis of the hip. Phys Ther. 2010;90:592-601. doi:10.2522/ptj.20090083.

2. Stensrud S, Roos EM, Risberg MA. A 12-week exercise therapy program in middle-aged patients with degenerative meniscus tears: a case series with 1-year follow-up. J Orthop Sports Phys Ther. 2012;42:919-31. doi:10.2519/jospt.2012.4165.

3. Garber CE, Blissmer B, Deschenes MR, Franklin BA, Lamonte MJ, Lee IM et al. American College of Sports Medicine position stand. Quantity and quality of exercise for developing and maintaining cardiorespiratory, musculoskeletal, and neuromotor fitness in apparently healthy adults: guidance for prescribing exercise. Med Sci Sports Exerc. 2011;43:1334-59. doi:10.1249/MSS.0b013e318213fefb.


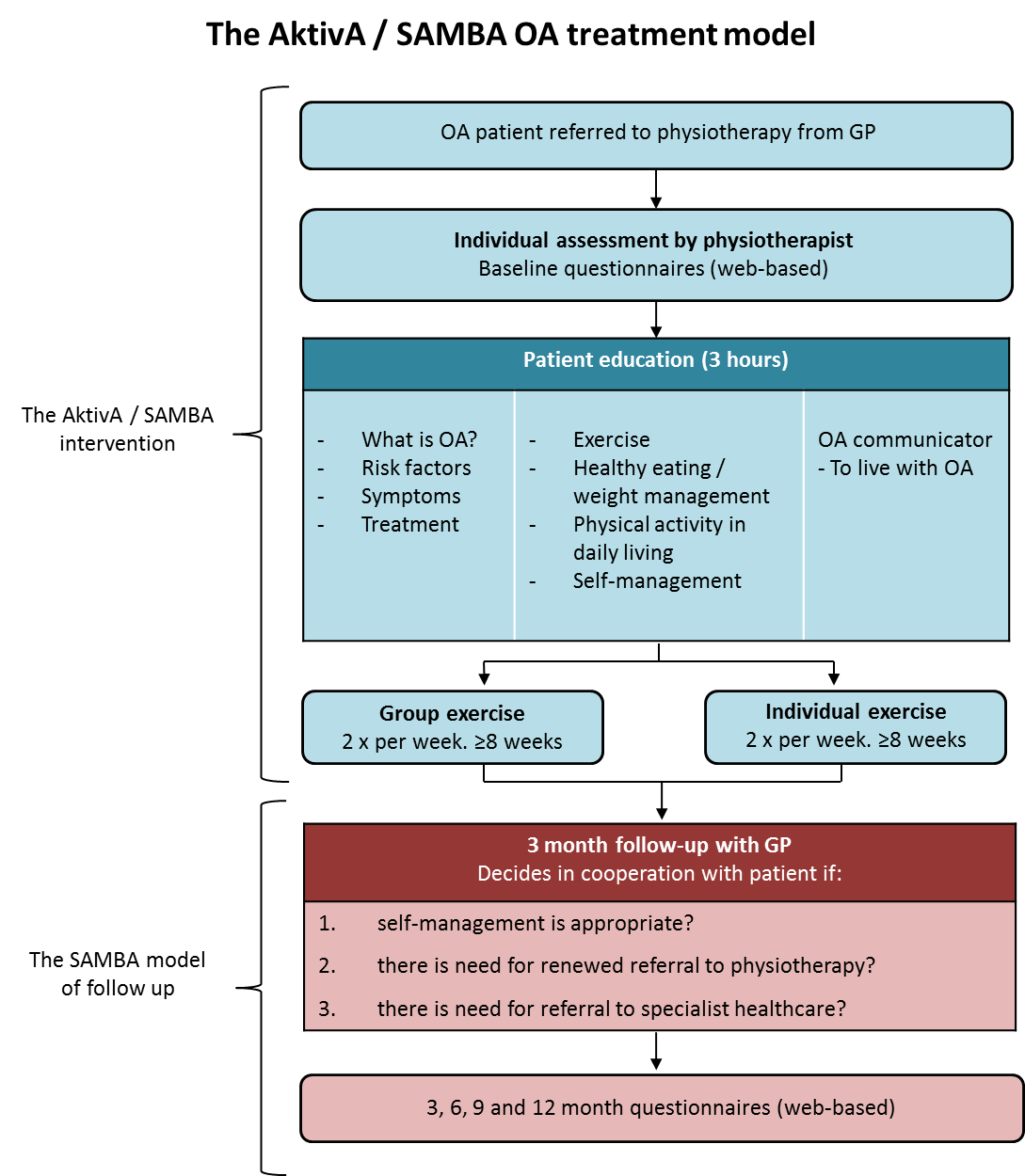


C

Exercises of the ActiveA program*


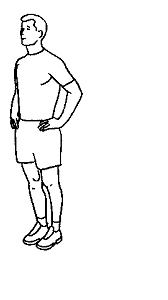

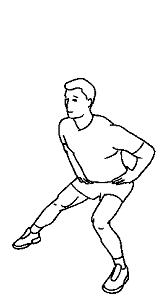

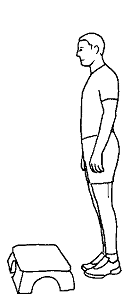

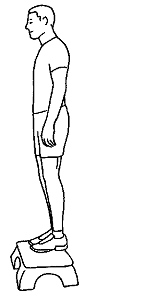

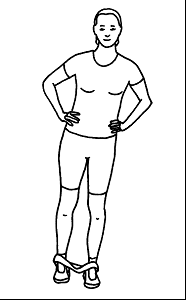

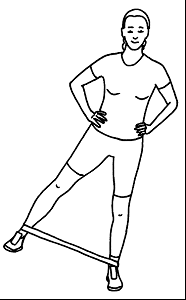

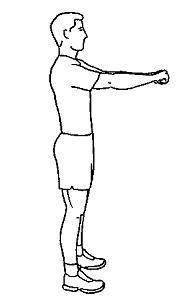

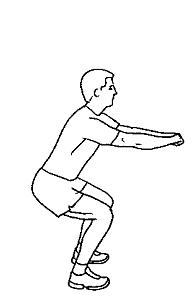

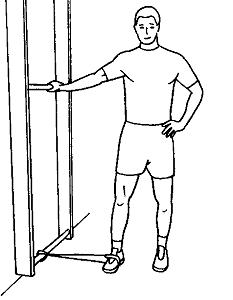

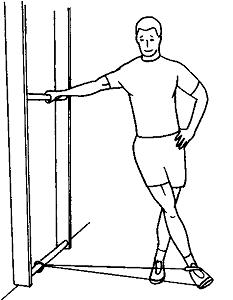

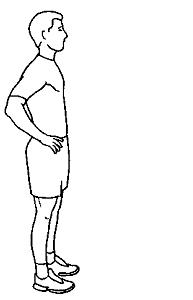

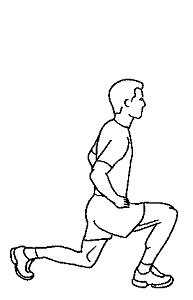

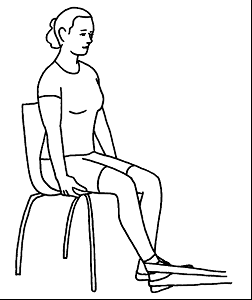

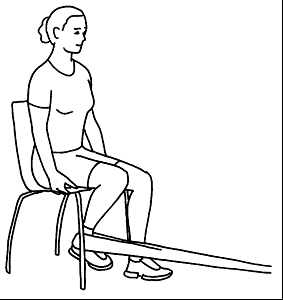


G

A

*Alternatives for progression of each exercise is not shown

B

D

E

F

Exercises for improving neuromuscular control and muscle strength


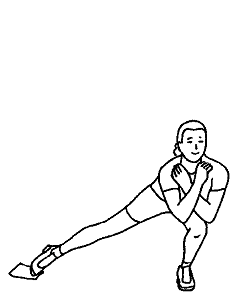

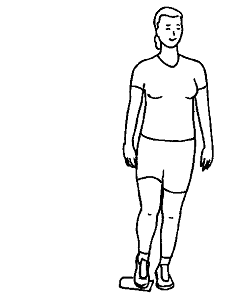

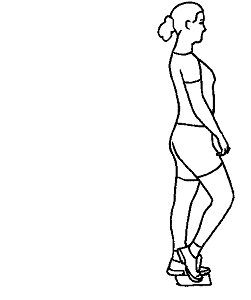

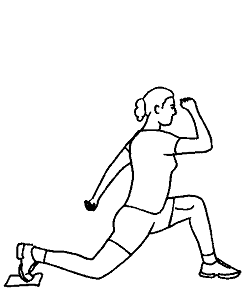

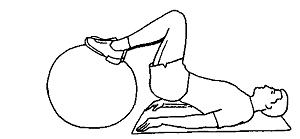

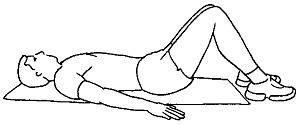

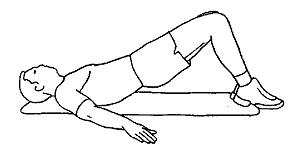

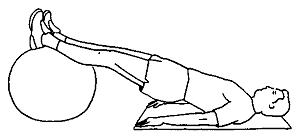

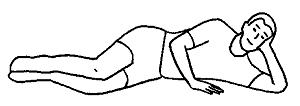

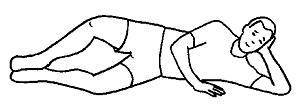

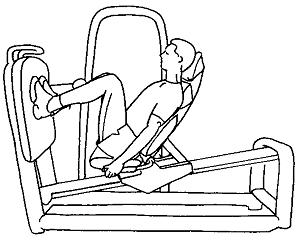

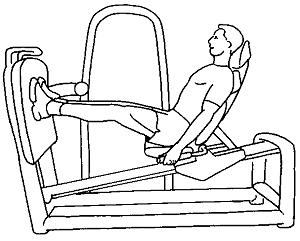

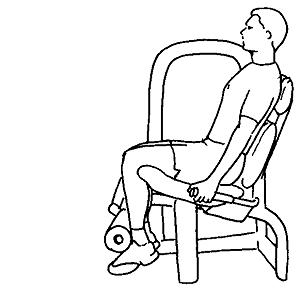

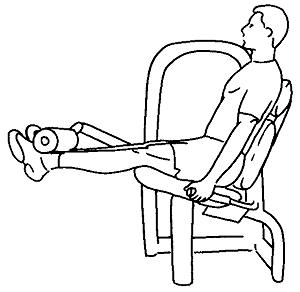

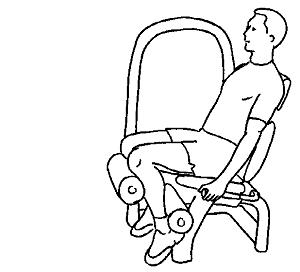

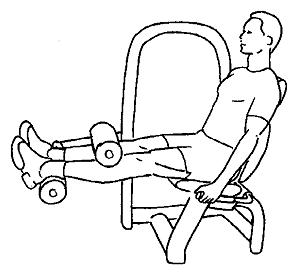


H

I

J

K

L

M

N

O


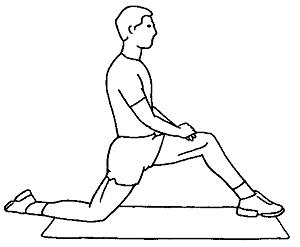

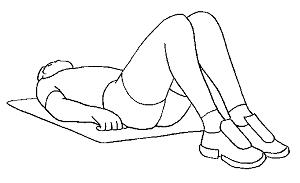

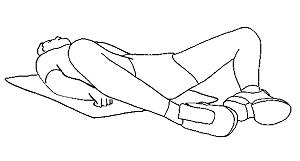

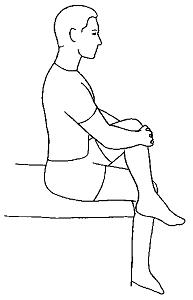

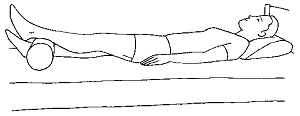


Exercises for improving range of motion

P

Q

R

S
